# Supplementary material for: Are carbon emissions trading and green financial instruments synergistic? -Comprehensive quantitative research based on content analysis
Source: PLoS One. 2024 Mar 7;19(3):e0298601. doi: 10.1371/journal.pone.0298601 (PMC10919601; doi:10.1371/journal.pone.0298601)
Supplement: S1 Data — (ZIP) [file pone.0298601.s001.zip › Data/地级市Z_F工作报告.zip!/╘╞─╧/╙±╧¬╩╨/└┤╫╘╣1⁄2╓┌║┼í╛┬φ┐╦╩2╛▌═°í┐_2018.docx]

各位代表：

现在，我代表市人民政府，向大会报告政府工作，请各位代表审议，请市政协委员提出意见。

一、过去五年的工作

第四届市人民政府履职五年来，在省委、省政府和市委的坚强领导下，在市人大及其常委会和市政协的监督支持下，认真贯彻落实党的十八大、十九大精神，坚持稳中求进工作总基调，践行新发展理念，统筹推进“五位一体”总体布局，协调推进“四个全面”战略布局，围绕全市经济社会发展“5577”总体思路，锐意改革创新，奋力攻坚克难，圆满完成“十二五”规划，启动实施“十三五”规划，经济社会发展迈上新台阶。

（一）经济实力大幅提升。五年来，我们始终把加快经济发展、提高发展质量作为全市工作的重中之重，深入开展“争先创优跨越发展”和“科教引领创新发展”大讨论大行动，认真落实稳增长各项政策措施，全市经济平稳较快发展。生产总值由2012年1000.2亿元增加到1415.1亿元，年均增长8.7%。发展质量和效益不断提高，人均生产总值突破9000美元，城乡居民人均可支配收入分别达34880元和13057元、居全省第二位；国地税收入分别完成337.5亿元和71.5亿元、总额居全省第二位，一般公共预算收入137.2亿元、居全省第三位；固定资产投资突破千亿元大关，达1080.8亿元，增长2.8倍；社会消费品零售总额367.4亿元，年均增长13.1%。经济结构不断优化，三次产业结构调整为10∶51.6∶38.4，非烟经济比重由61.6%提高到72.5%，民营经济比重由32.4%提高到36.4%，第三产业比重提高10.5个百分点。

（二）五网建设全面提速。五年来，我们始终把补齐基础设施短板作为头等大事，全力推进五网建设，基础设施条件全面改善。新增高速公路通车里程110公里，改扩建农村公路6274公里；玉磨铁路开工建设，玉蒙铁路、昆玉电气化铁路建成通车，玉溪进入高铁时代。5座通用机场规划建设加快，西南航空护林总站直升机场投入使用。大龙潭引水工程建成通水，13件重点水源工程竣工蓄水，新增库容2100万立方米，解决了57万农村人口饮水安全问题。完成4座水电站、7座风电场、5座光伏电站建设，新增电力装机64万千瓦，建成110千伏以上输变电站15座、城市燃气管道340公里。华为玉溪云计算数据中心投入运营，移动通信基站增加到1.4万座，通信光缆线路发展到5.4万公里，行政村光纤宽带和4G网络全覆盖。

（三）改革创新深入推进。五年来，我们始终把改革开放作为强大动力，实施创新驱动发展战略，发展动力活力明显增强。110项改革试点稳步推进，220余项改革举措逐步落地，创造了医改“玉溪模式”、殡改“玉溪经验”，深化改革走在全省前列。放管服改革获省肯定，下放64项市级审批权限，非行政许可事项全部取消，权责清单制度全面建立。提高沿湖县区生态建设考核权重，抚仙湖径流区实现统一托管。科技创新步伐加快，高新技术企业、专利授权量均居全省第二位，认定市级以上研发中心123户，科技对经济增长的贡献率达58.5%。对外开放步伐加快，累计引进市外国内资金3385亿元、外资1.8亿美元；进出口总额达21亿美元，比2012年增长2.9倍。

（四）城乡面貌明显改观。五年来，我们始终把统筹城乡发展作为重大任务，在提升规划水平上做文章，在完善基础设施上出实招，在强化精细管理上花功夫，在提高住房质量上求实效。推进“多规合一”，城乡规划体系不断完善。持续开展“六城同创”，抓好道路、供排水、地下综合管廊等市政基础设施建设，成功创建国家节水型城市，国家园林城市、卫生城市通过复查。江川实现撤县设区，县城提质扩容、特色小镇建设取得实效。建成公租房6.3万套，改造棚户区3.9万套、农村危房9.6万户。大力实施百村示范、千村整治，建成示范村93个、整治村952个。开展提升城乡人居环境行动，安装太阳能路灯5万盏，拆除临违建筑392万平方米，新建改建城乡公厕1037座，城市生活垃圾处理率达97%，城镇生活污水处理率达90%。29.6万农业转移人口成为城镇居民，城镇化率提高7.1个百分点。

（五）环境保护成效显著。五年来，我们始终坚持生态立市、环保优先，实施水体、大气、土壤污染防治行动计划，生态建设和环境保护迈出新步伐。坚持“五个坚定不移”，全面推进“四退三还”，投入84.8亿元实施119个三湖保护治理项目，抚仙湖水质稳定保持Ⅰ类、2016年全国水质良好湖泊考评排名第一，星云湖水质明显好转，杞麓湖水质由劣Ⅴ类改善为Ⅴ类，提前实现“水十条”国考目标。澄江、江川、华宁纳入国家重点生态功能区转移支付范围。治理水土流失面积1121平方公里，森林覆盖率达56.7%。节能减排任务全面完成，单位生产总值能耗累计下降38.3%，中心城区空气质量位居全国城市前列。

（六）民生福祉显著提升。五年来，我们始终坚持以人民为中心的发展思想，大力推进民生工程建设，人民群众获得感幸福感安全感进一步提升。全力打赢脱贫攻坚战，贫困发生率降至1%以下，有望实现全面脱贫攻坚目标。扶持创业就业，新增城镇就业12万人，转移农村劳动力65.4万人次。覆盖城乡居民的保障体系基本建立，各类社会保险参保人数达420万人次。公共服务体系不断健全，建成美丽校园160所，中小学D级危房全部消除，人均预期寿命提高到75.35岁，全部县区成为全国文化先进县，获评中国楹联文化城市，全民健身活动广泛开展。水库移民后期扶持工作取得实效。依法管理宗教事务，民族团结进步示范区创建成效明显。国防动员、国防教育不断加强，连续四届获评全国双拥模范城。加强安全生产，深化平安创建，严厉打击违法犯罪，社会保持和谐稳定，连续三届获评全国社会治安综合治理优秀市，荣获全国综治最高奖“长安杯”。妇女儿童、残疾人、老龄、红十字、关心下一代等事业健康发展，统计、外事侨务、应急管理、防灾减灾、人防、保密、档案等工作成效明显。

（七）自身建设不断加强。五年来，我们始终坚持依法行政，全面推进服务型政府建设，行政效能不断提升。扎实开展党的群众路线教育实践活动、“三严三实”和“忠诚干净担当”专题教育，推进“两学一做”学习教育常态化制度化，教育和引导政府机关工作人员增强“四个意识”，坚定“四个自信”，坚决维护以习近平同志为核心的党中央权威和集中统一领导。严肃整改中央第十一巡视组和省委第七巡视组巡视反馈问题。全面落实市委决策部署，自觉接受人大法律监督、工作监督和政协民主监督，办理人大代表建议1196件、政协提案1509件，办结率均达100%。全面加强法治政府建设，提请人大常委会审议制定地方性法规3部，清理废止政府规范性文件28件，健全重大行政决策程序和责任追究制度，市县区政府及工作部门法律顾问全覆盖。严格落实中央八项规定精神，解决“四风”突出问题，“三公”经费下降60%。行政监察、审计监督、政务督查不断加强。落实党风廉政建设主体责任和“一岗双责”，抓好政府系统廉政建设，深入开展反腐败斗争，严肃查处一批违法违纪案件。

刚刚过去的2017年，是本届政府履职的最后一年。我们认真落实市第五次党代会、市委五届二次全会和市四届人大五次会议精神，主要目标任务全面完成。生产总值增长9.3%，一般公共预算收入增长4.7%，固定资产投资增长20.9%，社会消费品零售总额增长12.5%，城乡居民人均可支配收入分别增长8.4%和9.1%，居民消费价格总水平上涨1.1%，城镇登记失业率3.3%，城镇化率50%，单位生产总值能耗下降3.2%。一年来，主要做了七个方面的工作：

一是全力以赴稳定经济增长。出台28项稳增长措施，制定落实加快民营经济、县域经济、园区经济发展的实施意见，整合财政资金6.9亿元、新增贷款90亿元支持实体经济发展，解决企业调头贷款6.8亿元，建立领导帮扶企业制度，开展稳增长促跨越百日攻坚，经济实现较快增长。加大开放型农业发展力度，蔬菜、林果、花卉、畜牧等产业量效齐增，跻身首批国家农业可持续发展试验示范区，农业增加值142亿元、增长6.3%，增速全省第一。着力打好工业转型攻坚战，卷烟产业止住下滑势头，现代装备制造、生物医药、电子信息等非烟产业加快发展，新增规上工业企业9户、高新技术企业13户，工业增加值662.5亿元、增长7.1%，非烟工业增加值增长22%。推进“四带多园”规划建设，甸中―十街农业生物产业园4个项目集中开工。启迪众创园投入运营，顺义产业园开园，九龙大数据产业园14个智能制造项目签约开工，园区工业增加值增长25.9%。玉昆、汇溪、福玉钢铁整合成为全省最大的民营钢铁企业，仙福、滇雪等10户企业上榜2017云南非公企业百强，民营经济增加值增长13.3%。易门、澄江、新平、元江入列省级县域经济先进县，易门生产总值过百亿元。推进全域旅游示范区创建，严厉整治旅游市场秩序，实现旅游总收入283.2亿元、增长73.9%。完善物流规划布局，中国西南·玉溪国际物流港启动建设，东南亚食品商贸仓储、九溪润特、传化通力等物流项目加快推进。深入开展十大扩消费行动，商贸、餐饮等传统服务业巩固提升，金融、信息等现代服务业加快发展，第三产业增加值543.7亿元、增长12%。

二是加快补齐基础设施短板。坚持重点项目副市长负责制和目标任务函告制，推行项目、任务、问题和责任清单管理，安排4.3亿元前期工作经费，争取5.3亿元省级重点项目投资基金，“四个一百”重点项目建设扎实推进。突出抓好145个五网项目建设，武易高速玉溪段、晋红高速、昆明东南绕城高速宜良至澄江段建成通车，弥玉、玉楚、江通、大戛、澄川、元蔓高速加快推进，新建改建农村公路2833公里，建制村公路硬化率100%、通客车率94%。玉溪至北京旅游列车、至郑州动车组开通，启动江川通用机场建设。15件重点水源工程、400件小坝塘除险加固进展顺利，建成农业高效节水减排面积20.8万亩，15.1万农村人口饮水安全得到巩固提升。建成4座电动汽车充电站，推广天然气用户6.6万户，中心城区气化率达62.3%。国家信息消费、宽带中国、智慧城市试点加快推进，云南联通玉溪数据中心主体工程完工，新建改造通信铁塔基站2478座。

三是强力推动重点领域改革。供给侧结构性改革成效明显，淘汰落后炼铁产能47万吨、水泥熟料138万吨、平板玻璃66万重量箱，取缔地条钢产能359万吨，销售商品房141.5万平方米，金融机构杠杆率下降0.68个百分点，为实体经济降低各类成本51.7亿元。放管服改革深入推进，承接、取消、下放、调整行政许可事项155项，清理规范行政审批中介服务事项91项，建立一站式惠民平台、办结时限缩短一半，“多证合一”、企业登记全程电子化和“双随机、一公开”监管全面实施。财税和投融资体制改革不断深化，整合设立国有资本运营公司、融资担保公司和9家专业化国有企业，与省再担保公司合作组建中小企业担保公司，设立省农业担保公司玉溪办事处，建立县乡财政增收激励机制。农村集体产权制度改革扎实推进，新增土地流转面积3万亩。城市管理和园区实体化改革稳步推进。列入全国首批殡葬综合改革试点和公立医院综合改革示范城市。

四是突出重点精准招商引智。出台21条招商优惠政策，聚焦珠三角、长三角、京津冀等重点地区，高位推进招商工作，外出招商180余次，组织开展“相约春天”“收获金秋”等重大招商引资引智活动，举办科教创新高峰论坛和全国3D大赛，获评2017年度中国最具投资吸引力城市。成功引进签约正威国际、华大基因、万科、复星、比亚迪、览海等知名集团，其中世界500强企业2户，引进市外国内资金912亿元、增长15%，外资1133万美元。与复旦、中山等15所知名院校签订合作协议，玉溪互联网大学、东南大学智慧城市研究院、同济大学智能制造研究院挂牌成立，新建院士工作站7个。深化与北京顺义、上海金山、江苏常州、广东佛山和惠州等城市友好合作。设立云南出入境检验检疫玉溪服务窗口，8户企业进入全国农产品出口百强，进出口总额增长4%。

五是全面推进城乡规划建设。启动新一轮城乡总规编制，完成美丽玉溪行动、试点县区“多规合一”等规划编制，列为全国城市设计和“城市双修”试点。科教创新城规划建设进展顺利，文化广播影视传媒中心等6个项目集中开工。城南客运站投入使用，市规划馆主体工程完工，建成地下综合管廊19.6公里、海绵城市项目58个。玉溪大河三期、高铁新城二期、老五街旧城改造工程启动，玉江大道改造提升、红龙路改扩建、新天地、红星国际广场、临岸三千城等项目有序推进。实施65个建制镇“一水两污”项目，乡镇供水、垃圾处理设施实现全覆盖，环境整治各项指标居全省前列。启动第二轮百村示范、千村整治。新平、元江获评国家园林县城，新平戛洒入选全国特色小镇，澄江广龙等6个小镇进入省级特色小镇创建名单。

六是着力解决突出环境问题。认真落实李克强总理重要批示精神和省委、省政府工作部署，坚决打好新时代抚仙湖保卫战，实施百日攻坚雷霆行动，100个突出问题已整改67个，启动径流区农业产业结构大调整，建立抚仙湖—星云湖绿色生态经济区。22户企事业单位和6122名农村居民退出抚仙湖一级保护区，径流区10万亩植被恢复项目开工建设，成功入选全国第二批山水林田湖草生态保护修复试点。3个湖泊国家湿地公园试点顺利推进，三湖“十三五”规划项目开工率达76%。全面推行河长制、湖长制，集中整治重点河流污染问题。加强大气污染防治，中心城区空气优良率达99.2%。启动土壤污染防治工作。完成营造林18.6万亩、低效林改造4万亩，治理陡坡地及石漠化面积7.9万亩。华宁、新平、峨山、元江获评省级生态文明县，13个乡镇获评省级生态文明乡镇。中央环保督察组反馈问题全部整改到位，省环保督察组反馈问题整改进展顺利。开展领导干部自然资源资产离任审计试点，强化环境执法监管，查处违法案件166件。

七是集中力量办好民生实事。全面实施七大民生工程，十件惠民实事圆满完成。扎实开展脱贫攻坚“找问题、补短板、促攻坚”专项行动和百日攻坚战，投入资金34.3亿元，发放小额信贷资金4.1亿元，实施易地搬迁5246人、危房改造8960户，3个贫困乡摘帽，17个贫困村出列，4万贫困人口脱贫。推进“就业创业玉溪”行动计划，新建创业孵化平台3个、众创空间4个，新增城镇就业2.8万人、农村劳动力转移就业21.5万人次。整合城乡居民医保，实现跨省异地就医结算，城乡低保标准每人每年分别提高到6072元和3420元。建成居家养老服务中心20个、乡镇敬老院5个，48.3万农户参加政策性农房地震保险。完成学前教育项目4个、“全面改薄”工程80个，玉溪衡水实验中学建成招生，在全省率先实现义务教育基本均衡。峨山、元江省级医养结合试点稳步推进，市医院改扩建、市儿童医院和8个县级公立医院补短板项目进展顺利。成功举办第五届中国聂耳音乐（合唱）周和云南省第十届民族民间歌舞乐展演，玉溪交通旅游广播开播，聂耳音乐之都建设成效显著。《云南省澄江化石地世界自然遗产保护条例》颁布实施。举办抚仙湖高原国际超级马拉松等赛事，玉溪籍运动员实现第十三届全运会金牌零突破。民族团结、信访维稳、禁毒防艾和反邪教工作进一步加强。

各位代表，过去五年，我们克服了市场需求萎缩、经济下行压力加大、烟草产业发展遇到波折、环境保护压力增加、补齐民生短板任务繁重等重重困难，全市经济社会发展取得显著成效。这是省委、省政府和市委坚强领导的结果，是市人大和市政协监督支持的结果，是全市人民凝心聚力、共同努力的结果。在此，我代表市人民政府，向全市广大干部群众，向市人大代表、政协委员、各民主党派、工商联、无党派人士和各人民团体，向中央和省驻玉单位、驻玉解放军和武警部队官兵，向各位离退休老同志，向所有参与和支持玉溪发展的各界人士，表示衷心的感谢和崇高的敬意！

各位代表！中国特色社会主义进入新时代，我国社会主要矛盾已经转化为人民日益增长的美好生活需要和不平衡不充分的发展之间的矛盾，我们充分认识到，在决胜全面建成小康社会、加快现代化建设征程中，玉溪发展不平衡不充分的问题也十分突出。发展不充分的表现是：烟草产业增势减弱，传统产业转型升级缓慢，新兴产业培育滞后，县域经济实力不强，增长动能不足，经济发展速度不快、总量不大、质量不高；基础设施不适应发展需要，重大项目进展慢，生产性投资不足，工业投资占比下降；财政收支矛盾突出，政府性债务化解和金融风险防控压力大。发展不平衡的表现是：城乡发展不协调，居民收入差距较大，农村发展滞后，基础设施薄弱；县域之间发展不平衡，红塔区经济总量占全市近一半，5个县区生产总值未过百亿元；社会事业发展滞后，基本公共服务供给不足，就业、教育、医疗、社保等方面仍然存在不少短板；生态保护与开发矛盾突出，三湖保护治理形势严峻，水体、大气、土壤污染防治任务繁重。与此同时，部分干部思想不够解放、干事创业精气神不足也是制约玉溪发展的主要障碍之一，有的干部站位不高、能力不足、作风不实、担当不够、创新意识不强，庸懒散混等为官不为现象不同程度存在。这些问题，我们必须采取有力措施加以解决，不断开创跨越式发展新局面，确保玉溪始终干在实处、走在全省前列。

二、今后五年的基本思路和目标任务

今后五年，是玉溪全面建成小康社会的决战决胜期、加快产业转型升级的攻坚期、实现跨越式发展的机遇期。我们一定要认真贯彻党的十九大精神，以习近平新时代中国特色社会主义思想为指导，按照省委、省政府对玉溪的新定位新要求，坚持稳中求进工作总基调，坚持新发展理念，坚持加快发展与高质量发展并重，统筹推进“五位一体”总体布局，协调推进“四个全面”战略布局，以深化供给侧结构性改革为主线，坚持经济社会发展“5577”总体思路不动摇，全面落实市委关于深入学习党的十九大精神促进玉溪跨越式发展的决定，把支持传统产业优化升级、培育壮大新兴产业、加快实体经济发展作为重大战略任务，持续用力，久久为功，彻底扭转发展动能不足、发展速度不快的被动局面，努力将市第五次党代会描绘的宏伟愿景变为现实场景。

主要目标建议为：生产总值突破2300亿元、年均增长8.5%以上，固定资产投资突破2500亿元、年均增长18%以上，一般公共预算收入达165亿元、年均增长4%以上，社会消费品零售总额达650亿元、年均增长12%以上，城乡居民人均可支配收入分别突破5万元和2万元、年均增长8%和9%以上，城镇登记失业率控制在4%以内，城镇化率达58%。

今后五年要突出以下七项重点任务。

（一）扭住产业培育不放，奋力实现经济追赶跨越。加快产业培育发展，是实现经济追赶跨越的关键。我们要坚持产业富市战略不动摇，抢抓新一轮科技革命和产业变革孕育兴起、省委省政府加快八大产业发展的重大机遇，主动融入国家和省的发展战略，加快构建实体经济、科技创新、现代金融、人力资源协同发展的现代产业体系。围绕“三区一港”发展定位，把发展实体经济作为追赶跨越的重要支撑，“四带多园”作为经济增长的重要平台，技术创新作为转型升级的重要动力，集中精力和资源要素，一手抓传统产业巩固提升，一手抓新兴产业培育，把现代装备制造、生物医药及大健康、电子信息、现代物流、文化旅游产业培植成新的支柱产业，七大产业增加值占生产总值的70%以上。持续打好民营经济、县域经济、园区经济三大战役，2020年所有县区生产总值突破百亿元，玉溪高新区产值超千亿元，打造一批特色鲜明、竞争力强的产业集群，民营经济占生产总值的45%以上。

（二）聚力夯实基础设施，不断改善持续发展条件。体系完备的基础设施是实现追赶跨越的重要前提。我们要抢抓国家实施区域协调发展战略和省委、省政府加快五网建设的重大机遇，坚持不懈加强水利、铁路、公路、航空、管道、电网、信息、物流设施建设，形成内通外畅、支撑有力的现代基础设施网络体系。加快综合交通设施建设，玉磨铁路建成通车，启动建设呈贡—澄江—江川—红塔区城际铁路，新增高速公路800公里，改造国省道1300公里、县乡道1400公里，全部自然村通硬化路。建成5个通用机场。加快骨干水源和民生水利工程建设，实现安全饮水全覆盖，配合推进滇中引水工程，新增蓄水库容1亿立方米。加快骨干电源、城乡电网和油气配套设施建设，构建清洁高效、安全低碳的现代能源体系。加快互联网出口带宽、物联网、4G基础设施改造提升，启动5G设施建设，固定宽带家庭普及率达80%以上，建成面向南亚东南亚的通信枢纽和区域信息汇集中心。科学布局现代物流园区，建成区域性国际农产品交易中心和中国西南·玉溪国际物流港。

（三）紧紧依靠创新驱动，不断提高发展质量和效益。创新是引领玉溪追赶跨越的第一动力。我们要坚持创新活市战略不动摇，加大资源整合力度，加快构建“一核五片七区”的科教创新城空间格局，推动产城融合、产教融合、人才聚合，成功创建国家创新型城市，基本建成滇中科教创新中心。加快引进一批知名高校和科研院所，建设与主导产业相配套的院士工作站、重点实验室、企业技术中心。在烟草、花卉、水环境治理、生物医药等领域发力，引进消化一批、研发创新一批、引入高端人才带入一批，形成一批具有自主知识产权的科技成果并加速转化运用。稳定增加科技投入，建立以企业为主体、市场为导向、产学研深度融合的技术创新体制，高新技术企业达150户以上。强化专业技术人才、高技能人才培养引进，打造领军人才和创新团队，形成大众创业、万众创新的良好氛围。到2022年，研发经费投入占生产总值2.5%以上，科技进步对经济增长的贡献率达60%以上。

（四）敞开开放合作大门，倾力加快外向型经济发展。改革开放是推动追赶跨越的强大动力。我们要坚持开放兴市战略不动摇，抢抓国家推进“一带一路”建设、云南加快建设面向南亚东南亚辐射中心的重大机遇，充分发挥区位优势，创新开放合作方式，强化与南亚东南亚国家之间的经济、人文、技术等交流合作，主动参与孟中印缅经济走廊建设，加快构建对外开放合作新格局，基本建成辐射南亚东南亚的重要物流枢纽。坚持不懈把招商引资引智作为经济工作的生命线，全面加强与珠三角、长三角、京津冀等区域合作，主动融入滇中城市经济圈，围绕重点产业、盯住知名企业抓招商，提高招商引资质量。优化出口产品结构，巩固优质农产品出口优势，加快九龙、龙泉片区智能终端制造产业园建设，扩大工业产品出口，确保外贸进出口总额突破40亿美元。深化重点领域和关键环节改革，以改革促开放，以开放倒逼改革，坚决破除一切不合时宜的思想观念和体制机制障碍。

（五）始终坚持绿色发展，全面提升可持续发展能力。生态环境是重要的公共产品，环境保护是政府的重要职责。我们要坚持生态立市战略不动摇，践行绿水青山就是金山银山的理念，实施清水、净土、蓝天、国土绿化和城乡人居环境提升五大行动，着力解决突出环境问题，成为全省生态文明建设排头兵。坚决打赢新时代抚仙湖保卫战，落实河长制、湖长制，完成抚仙湖、星云湖一级保护区2.2万生态移民搬迁，强化三湖流域生态系统修复，落实土地休耕轮作规划，调整优化农业生产结构，确保抚仙湖水质稳定保持Ⅰ类，力争星云湖水质达到Ⅳ类，杞麓湖水质稳定保持Ⅴ类。加强生物多样性保护，加快森林玉溪建设，25度以上坡耕地逐步退耕还林还草，森林覆盖率达60%以上。落实主体功能区规划，建立差异化绩效考核评价机制，优化空间开发格局，推动生产生活方式绿色低碳发展。实行最严格的生态环境保护制度，严守生态红线，坚决制止和惩处破坏生态环境行为，守住绿水青山、蓝天白云。

（六）统筹城乡协调发展，着力加快美丽家园建设。城乡融合发展是加快玉溪现代化建设的内在要求。我们要坚持新型城镇化和乡村振兴两手抓，不断增强城乡区域发展的协调性。大力实施乡村振兴战略，按照产业兴旺、生态宜居、乡风文明、治理有效、生活富裕的总要求，深化农村产权制度改革，落实土地承包到期后再延长30年的政策，完善承包地“三权”分置制度，激活农村发展活力。推动基础设施向农村拓展，改善农业农村发展条件；加快培育现代农业产业体系，推动烤烟、蔬菜、花卉、林果、畜牧等特色产业提档升级，扩大农产品精深加工，发展乡村旅游，促进一二三产业融合发展，拓宽农村居民就业和增收渠道；推动公共服务向农村延伸，创新乡村治理方式，补齐农村社会建设短板，加快农业农村现代化进程。坚持走以人为核心的新型城镇化道路，积极构建“一核、双心、两轴、四城、多节点”的城镇化空间布局框架，高起点规划、高标准建设、高水平管理，完善城镇体系规划，加快中心城区“增绿添色、点亮玉溪、六城同创、建设花城”4大工程建设，推进县城提质扩容，抓好特色小镇建设，促进农村人口就近就地市民化。到2022年，把玉溪建成全省区域性中心城市和创新开放生态宜居文明幸福的魅力之城。

（七）强化创新社会管理，提速补齐民生领域短板。解决民生问题始终是政府工作的出发点和落脚点。我们要坚持共享和市战略不动摇，始终把人民对美好生活的向往作为奋斗目标，努力让各族群众幼有所育、学有所教、劳有所得、病有所医、老有所养、住有所居、弱有所扶。在巩固提升脱贫成果上下功夫，确保工作力度不减、支持政策不变，扶贫与扶志、扶智相结合，实现贫困群众稳定脱贫、贫困地区可持续发展。落实积极就业创业政策，实现高质量充分就业。实施全民参保计划，让人人享有基本社会保障。建立多主体供给、多渠道保障、租购并举的住房制度，基本消除城市棚户区和危房，修缮加固农村C级危房、全面消除D级危房。优先发展教育事业，持续改善办学条件，中心城区新建迁建幼儿园14所、小学3所，新建初中4所、高中1所，基本满足市民的优质教育需求，率先在全省实现教育现代化。深入推进健康玉溪建设，健全基层医疗卫生服务体系，深化公立医院改革，改善县级办医条件，提升乡镇卫生院医疗水平，加快培养全科医生，下决心解决看病难看病贵问题。加快老龄事业和产业发展，有效应对人口老龄化。大力弘扬“玉汝于成、溪达四海”的玉溪精神，深入实施公民道德建设工程，广泛开展群众性精神文明创建活动，推动文化持续繁荣。全面贯彻党的民族政策和宗教工作基本方针，争创全国民族团结进步示范市。树立安全发展理念，全面推进法治玉溪、信用玉溪、平安玉溪建设，打造共建共治共享的社会治理格局。

三、2018年重点工作

今年是贯彻党的十九大精神的开局之年，是决胜全面建成小康社会、实施“十三五”规划承上启下的关键一年。做好今年政府工作，要以习近平新时代中国特色社会主义思想为指导，全面贯彻中央经济工作会议、省委十届四次全会、市委五届五次全会精神，坚持稳中求进工作总基调，坚持经济社会发展“5577”总体思路，以供给侧结构性改革为主线，全面推动质量变革、效率变革、动力变革，统筹推进稳增长、促改革、调结构、惠民生、防风险各项工作，全面落实重大风险防范、巩固提升脱贫成果、强化污染防治三项重点任务，促进经济社会持续健康发展。

经济社会发展目标建议为：生产总值增长8.5%，固定资产投资增长20%，一般公共预算收入增长3%，社会消费品零售总额增长12%，城镇居民人均可支配收入增长8%，农村居民人均可支配收入增长9%，居民消费价格涨幅控制在3%以内，城镇登记失业率控制在4%以内，城镇化率达51%，单位生产总值能耗完成省下达目标。

围绕目标任务，着力抓好十个方面的工作。

（一）在供给侧结构性改革上下足功夫，努力实现高质量发展

供需发力优化供给。在提高供给质量上着力，不断转变经济发展方式，变要素驱动为创新驱动，变投资拉动为投资和消费双拉动，变工业带动为工业和服务业双带动。全力打好“绿色能源、绿色食品、健康生活目的地”这三张牌，增强发展新动能。紧盯七大产业精准施策，完善产业发展规划，建立产业项目库，明确年度施工图，确保每个产业都有一批企业、项目接续建设，推动产业向开放型、创新型和高端化、信息化、绿色化转型发展。在增加有效投资上着力，强化项目前期准备、申报、储备，扎实推进“四个一百”重点项目和“三个一百”工业转型升级项目建设，优化投资结构，确保非电工业投资增长20%以上。在挖掘消费潜力上着力，全面实施服务经济倍增计划，建立部门联席会议制度，制定年度实施方案，促进服务业提档升级，努力释放消费需求，确保第三产业增加值增长10%以上。在提高发展质量上着力，实施质量提升行动，鼓励和支持企业加快技术创新，推广降本增效技术，培育开发名牌产品，提高企业经济效益。推进农业供给侧结构性改革，以做优做强绿色食品为重点，持续提升农业有效供给。

深入推进三去一降一补。综合运用市场化法治化手段化解过剩产能，严禁新上产能过剩项目，严防“地条钢”死灰复燃，淘汰炼铁产能55万吨、水泥熟料45万吨、铜冶炼5万吨。调节住房供需结构，销售商品房150万平方米，严控房地产库存增长。严格规范政府性债务限额和预算管理，健全风险管控和应急处置机制，积极稳妥化解潜在风险。严厉打击金融诈骗、非法集资等违法违规金融活动，严防发生系统性区域性风险。严格落实减税降费政策，开展涉企收费专项检查，降低企业制度性交易、用地、用能和物流成本，激发市场主体活力。进一步补齐基础设施、生态环保、公共服务、民生保障等方面的短板。

强化经济运行调节。加强宏观经济形势分析研判，把握好国家继续实施积极的财政政策导向，充分发挥综合经济部门和市属国有企业的积极性，盯住投资重点精准包装项目，争取一批重大基础设施项目进入省级以上PPP项目库，一批重大产业项目得到上级财政支持。把握好国家实施稳健中性的货币政策导向，不断创新融资方式，通过政府和企业发债、股权和资产变现、股市融资等多种方式筹集资金，解决好重大项目建设资金需求。制定实施促进经济持续健康发展的政策措施，建设规上项目在线监测系统，强化项目审批、用地、投资等协调调度，确保续建项目不停、新上项目及时启动。突破用地障碍，压实县区耕地占补平衡责任，新增耕地5000亩、建设用地4500亩。突破企业融资障碍，盘活闲置土地资产，组建玉溪投资控股集团，建立政策性融资担保风险分担机制，充分发挥产业基金的带动作用，强化银政银企合作，引导金融机构回归本源，支持实体经济加快发展。加强经济运行预警监测，做好第四次全国经济普查。

（二）在工业转型升级上下足功夫，加快培育非烟支柱产业

锁定重点产业。实施中国制造2025玉溪行动计划，落实工业产业链拓展延伸实施意见，依托工业投资公司多渠道融资20亿元，建立产业发展基金，支持重点产业发展，加快173个工业项目建设，力争67个按期开工、62个竣工投产，千方百计稳存量、扩增量、提质量，确保工业增加值增长7.6%以上。支持传统产业优化升级、提质增效。围绕红塔集团原料需求，压实县区烤烟生产责任，落实种植面积、种植品种，确保完成收购任务；支持企业保总量、调结构、拓市场、去库存、增效益，推动配套产业二次创业，营造产业发展浓厚氛围，发挥烟草“稳定器”作用。抢抓矿冶行业市场复苏的有利时机，深化与昆钢集团合作，支持原料采购和品牌整合；引导重点企业加快产能置换、开发钢制品，实现转型升级，确保矿冶产业增加值增长15%以上。支持新兴产业成长壮大。加快九龙、龙泉片区智能终端制造产业园标准化厂房建设，确保已落户企业入园生产。加大对蓝晶科技的扶持力度，加速年产3500万片LED衬底片项目投产，形成4800万片生产能力，建成全国最大的蓝宝石基地。围绕建设制造业强市目标，制定数控机床、新能源汽车及配套产业发展规划，加快数控机床产业聚集发展，逐步达到3万台生产规模，建成全国最大的生产基地。加快新海宜新能源汽车及配套、通用航空装备、装配式建筑、电梯、云轨等项目建设，实现装备制造产业增加值增长10%以上。加快生物医药产业园核心区建设，支持沃森、维和、九州生物等企业加快核心产品产业化，生物医药产业增加值增长20%以上。

锁定重点园区。认真落实加快园区经济发展的实施意见，强力推进实体化改革，研和工业园区收回市管、委托玉溪高新区管理，完成其他5个省级工业园区人权、事权、财权制度改革，让园区具有独立的融资权。支持引入战略投资者建设生物医药、智能制造、装备制造、跨境贸易加工等园中园，完成园区基础设施投资100亿元，建成标准化厂房80万平方米，研和、通海园区污水处理厂建成运营。高新区新增4个投资10亿元以上、15个投资1亿元以上项目，红塔、大化、易门工业园区各新增3个投资1亿元以上项目，其他工业园区各新增3个投资5000万元以上项目，园区增加值增长10%以上，高新区非烟生产总值突破百亿元。

锁定重点企业。出台扶持培育重点骨干企业发展的政策措施，确定100户成长性好、税收贡献大、吸纳就业多的重点企业优先扶持，通过战略重组、技术创新等方式发展壮大。加快发展民营经济，实施民营企业转型升级、互联网+小微企业、中小企业管理提升3大行动计划，强化金融、人才培训、管理咨询等服务，培育小巨人企业50户，新增规上企业20户、省级成长型企业15户、新三板挂牌企业1户，民营经济增加值增长13%。弘扬企业家精神，保护企业家权益，激发企业家创业热情。

（三）在现代服务业发展上寻求突破，努力推动第三产业迈向中高端

全力打造文化旅游和大健康产业。围绕打造全国一流的“健康生活目的地”目标，以产品开发为主线、大项目建设为抓手，优化“一核两翼”规划布局，做大做强文化旅游和大健康产业。大力改善旅游基础设施条件，加快38个续建、10个景区提档升级项目实施，推进澄江、新平全域旅游示范区创建，擦亮抚仙湖、帽天山、花腰傣三张名片，确保旅游总收入增长16%以上。提速寒武纪乐园、广龙旅游小镇、仙湖山水等项目规划建设，推进抚仙湖国家级旅游度假区创建工作。完成高鲁山、龙马山生态休闲文化旅游园前期工作，确保青花街等项目建成运营。推进新平磨盘山国家森林公园、元江果香四季国际旅游度假区建设，建好哀牢山―红河谷民族文化旅游区。加快陶、铜、银、刺绣等特色旅游商品开发。积极发展智慧旅游，重拳整治市场秩序，提升旅游整体形象。围绕把澄江建成“国际旅游城市、国际健康养生城市、国际会议中心城市”目标，落实“一城五镇多村”规划，按照国际一流标准建设云南抚仙湖国际医疗健康城、南亚东南亚医学中心及生命健康城，加快前期工作，尽快开工建设，将玉溪打造成为世人向往的“健康生活目的地”。

做大现代物流产业。出台促进现代物流产业发展政策措施，完善物流信息平台，力争培育2家2A级以上物流企业，推进中国西南·玉溪国际物流港、通海杨广冷链物流园建设，加快传化通力公路港二期、江川雄关和元江甘庄农产品物流园、昆玉铁路货运场前期工作，启动国际农产品交易中心、活发物流、滇中玉溪粮食物流产业园建设，确保九溪润特物流一期、中国东南亚食品商贸仓储物流港一期竣工，支持嘉德集团发展跨境贸易，与云南宝象集团合作建设综合物流基地，现代物流业增加值增长15%以上。

提升传统商贸流通业。优化商业消费网点布局，落实“吃在玉溪、购在玉溪”三年行动，加快中心城区电子商务示范产业园、特色餐饮街区、商业区建设。推进国家电子商务示范城市建设，搭建农村电子商务公共服务体系，完善农村消费网络，完成6个农贸市场和10个乡镇加油站点建设。组织实施一批现代服务业示范项目，新增一批限额以上商贸企业。

培育发展金融服务业。编制玉溪金融中心规划，加快建设商业金融核心区，扩大本地金融机构规模，积极引进金融机构和证券公司，创新服务产品，发展普惠金融，新增贷款增长5%以上。开发重点产业保险产品，充分发挥保险保障功能。

（四）在落实乡村振兴战略上寻求突破，努力推动农业农村发展再上新台阶

制定乡村振兴规划。把实施乡村振兴战略作为新时代做好“三农”工作的总抓手，认真落实中央实施乡村振兴战略的意见，抓住财政优先保障和金融重点倾斜机遇，科学制定战略规划，积极争取省级试点，推动农业全面升级、农村全面进步、农民全面发展。

巩固脱贫攻坚成果。坚持政策不脱、帮扶不脱、责任不脱、监管不脱，突出产业发展增收、人居环境整治、社会保障巩固三个重点，制定巩固提升脱贫成果实施方案，建立健全防止返贫机制。以哀牢山区、革命老区、少数民族地区为重点，强化因人因户精准措施落实，着力解决贫困分散人口脱贫问题，确保1.2万贫困人口脱贫，不落一户、不掉一人。

加快特色产业发展。围绕打造“绿色食品牌”，制定实施“一村一品、一县一业”方案，发展大产业、培育新主体、建设新平台，确保农业增加值增长6%。种植粮食160万亩，总产6亿公斤，提高粮食供给保障能力。调整优化抚仙湖周边种植结构、新增烤烟1万亩，全市种植55.6万亩、收购烟叶150.1万担，巩固云烟之乡地位。种植蔬菜130万亩、鲜切花3.5万亩、生物药原料6.5万亩，新增水果2万亩。制定实施红河谷―绿汁江热区开发计划，加快基础设施建设和土地开发整理，打造热区特色农业发展新的增长极。启动国家农业可持续发展试验示范区11个项目建设。加快亚洲花卉科创谷建设，推动爱必达公司上市。优化养殖业布局，发展规模化养殖，抓好华西希望德康集团新平种猪场建设，建成生猪家庭农场100个，新建云岭牛养殖示范场5个，畜牧业产值突破100亿元。积极培育新型农业经营主体，启动现代农业产业园创建，新增市级龙头企业5户、市级家庭农场50个、农业专业合作社20个以上，培育销售收入5亿元以上农业小巨人5户。大力发展农产品精深加工，加工业产值达280亿元。加快农业标准化、品牌化、市场化步伐，建成市农产品质量安全检验检测中心，在一线城市设立农产品销售中心，新增“三品一标”农产品10个、云南名牌农产品3个，确保绿色食品产业发展走在全省前列，成为玉溪的新名片。

建设美丽文明乡村。深化农村土地制度改革，全面完成农村土地承包经营权确权登记颁证，加快推进农村土地征收、集体经营性建设用地入市、宅基地制度改革试点。推进农村集体产权制度改革，完善农业支持保护制度。开展农村人居环境百日攻坚，每个县区抓好10个示范村建设，村庄生活垃圾处理率达95%以上。推进以直过民族为重点的第二轮百村示范、千村整治，建成示范村24个、整治村29个。完成中低产田地改造13.8万亩，新建改造10千伏及以下农网261公里，巩固提升15万农村人口饮水安全。加强农村基层基础工作，开展农村社区建设试点，创建法治乡村、平安乡村，提高乡村德治水平。传承优秀文化，抓好28个传统村落保护。

（五）强化基础设施网络建设，破解跨越发展瓶颈制约

加快综合交通基础设施建设。推进弥玉、玉楚、澄川、元蔓4条高速公路建设，确保江通、大戛高速大开门至新平县城段、弥玉高速华宁试验段建成通车，实现县县通高速目标。力争永金高速新平至元江段、机场高速红塔区至江川段开工建设，配合昆明市开工建设晋易高速，启动澄华、新平大开门至石屏宝秀、新平戛洒至镇沅者东3条高速公路前期工作。拆除江华路、江通路收费站。完成国道213线改造，改造提升国省道5条412公里、县乡道4条131公里，推进“四好农村路”建设，改扩建农村公路2000公里，建制村通客车率100%。完成公路投资171亿元。配合推进玉磨铁路建设，争取呈澄城际轨道交通试验段、比亚迪·玉溪云轨示范线启动建设。加快江川通用机场建设，推进澄江、华宁、新平、元江通用机场前期工作。

强化水利和能源设施建设。开工建设新平洋发城中型水库和元江陆家店、华宁小箐、通海琉璃河小（一）型水库，加快13件水源续建工程、大龙潭引水通海支线、澄江甸垛龙潭调水工程建设，推进新平白沙河中型水库和“西水东调”工程前期工作，完成投资23亿元。抓好220千伏永济输变电、应急气源储备中心、油气管网红河支线等工程建设。

提速信息基础设施建设。扩充市县光纤骨干网络容量，建设1000座物联网基站，推动新一代公众移动通信、物联网运用。加快城市大数据运营中心建设，推动一站式信息惠民服务平台运用。启动信息安全产业基地建设，促进云产业基地形成初步规模。

（六）强化创新驱动，推动科教创新城落地见效

进一步强化科技创新。加大财政投入，支持企业自主研发，力争研发经费占生产总值比重提高到1.05%。发挥玉溪启迪众创园、云科高新众创空间等平台作用，积极申报国家和省级“双创”示范基地。新增院士工作站3个、市级重点实验室和工程技术中心5个，申报高新技术企业20户以上、省科技型企业10户以上。加强知识产权保护，积极推进国家知识产权质押融资试点。实施“兴玉英才计划”，完善激励机制，大力培养和引进各类高层次人才和创新团队，推动创新精神、企业家精神和工匠精神融合，激发创新创业活力。

加快推进科教创新城建设。加大引智引校力度，建立科教创新投资基金，组建科教创新城管理委员会，启动土地一级开发整理，完成核心区11条道路建设。加快医养中心、体育运动中心建设，完成玉溪卫校迁建。推进科技创新研发中心、创新创业园建设。制定招商引智优惠政策及项目准入制度，加快已签约高校合作项目落地，推进与北京航空航天大学、北京体育大学等高校的合作。

（七）强化改革开放，努力拓展对外合作空间

深化重点领域改革。持续推进放管服改革，全面实施市场准入负面清单制度，进一步清理规范中介服务，积极推进综合监管和检查信息公开，优化营商环境。发挥投资项目审批服务中心作用，加快推进并联限时审批，大幅精简审批环节、压缩办理时限。大力推进“互联网+政务服务”，提高服务便利化水平，让群众办事少跑腿，努力实现“最多跑一次”。深化投融资体制改革，做强融资担保公司，做大市属专业投资公司，建立健全投融建管营一体化体制机制。深化财税体制改革，整合财政专项资金，优化支出结构，压缩一般性支出，积极稳妥推进财政事权和支出责任划分改革，完善县乡财政管理体制。加快公立医院改革，推进三湖管理体制、统计管理体制、价格体制、事业单位分类、自然资源资产管理等改革。

加大招商引资力度。健全招商工作机制，成立招商引资工作委员会、各产业招商组、项目落地建设要素保障组和外资招商工作组，建立专家咨询评估制度，持续强力推动招商引资工作。锁定重点区域和行业龙头企业精准招商、以商招商、委托招商，力争引进一批大项目好项目。办好“相约春天”“收获金秋”等招商活动，策划包装100个以上重大项目。落实跟踪督办制度，强化项目落地考核，加快185个已签约项目落地，确保实际利用市外国内资金1000亿元、增长11%，外资1500万美元、增长32%。

提高开放合作水平。制定支持企业扩大出口的政策，加快外向型生产基地建设，设立对外贸易服务中心，改造提升国外农产品销售网点，培育一批跨境电子商务平台和企业。争取昆明海关设立玉溪服务窗口，扩大本地农产品出口，提高生物制药、智能制造等工业产品出口比重，力争手机出口3亿美元以上，确保外贸进出口总额增长10%以上。深化与友好城市之间的合作，加强与滇中各州市产业、交通、环境等方面的对接，积极融入滇中城市经济圈建设，加快推进昆玉一体化。

（八）强化新型城镇化建设，努力提升辐射带动能力

提升规划管理水平。完成城乡总体规划修编，完善中心城区控制性详细规划和城市建筑风貌导则，做好城市设计试点，构建“多规合一”协调机制和一张蓝图管控体制。抓好县区控规编制，实现江川区控规一张图，做好50个省级示范村规划编制，完成市规划馆建设。健全规划执行监督体系，建立乡村规划建设专管员制度，提高农村建房规划许可覆盖率。

提升建设管理水平。深入推进“六城同创”，做好中国人居环境奖申报工作，建成20.9平方公里海绵城市试点项目，加快智慧城市建设。推进“城市双修”，加快“增绿添色”和“花城”景观项目建设，抓好50公里城市地下综合管廊建设，启动第三污水处理厂、北片区供水工程，建成排水管网16公里，开工12条市政道路和城北客运枢纽建设，完成玉江大道、东风路、新西河路、晋红高速连接线等改造提升。优化地下综合管廊、海绵城市和市政道路等项目施工方案，强化工期管理，科学组织建设，最大限度减轻对群众生产生活的影响。推进城市“四治三改一拆一增”综合整治，抓好金水河等4条黑臭水体治理，完成玉溪市污水再生利用、餐厨垃圾综合处理、生活垃圾焚烧发电等项目建设，提标改造县城污水处理厂，改造棚户区4000套、农村危房4000户。深入开展“厕所革命”，新建改造城市公厕57座、旅游厕所72座、农村公厕150座。抓好澄江撤县设市，推进江川区与红塔区协同发展，加快澄江、峨山县城提质扩容。申报1个国家园林县城，加快6个特色小镇建设，省级特色小镇完成总投资60%以上。

提升城镇管理水平。全面启动城市管理执法体制改革，成立市、县区城市管理委员会，综合设置县区政府城市管理机构。实施城镇绿化条例。出台违法建筑认定标准指导意见，依法清理存量违法违规建筑。严格执行城市管理条例及配套办法，提高精细化管理水平。开展城市重要节点和交通环境综合整治，改善城南客运站周边交通条件，缓解交通拥堵。加强停车场规划建设，缓解医院学校周边等重点路段停车难问题。深化户籍制度改革，全面落实转户居民社保、就业、教育、住房等权益，力争4万农业转移人口有序实现市民化。

促进房地产和建筑业健康发展。加快建立“租购并举”的住房制度，力争启动中心城区利用集体建设用地建设租赁住房试点，支持金融机构推出租房贷款。规范建筑市场秩序，积极推广装配式建筑、绿色建筑。加快培育龙头骨干企业，扶持本地建筑企业做大做强，做好省建投六公司入驻玉溪服务工作，吸引更多建筑企业入驻玉溪。完成房地产业投资200亿元以上，建筑业增加值增长25%以上。

（九）强化三湖保护治理，努力建设美丽玉溪

加强三湖水污染治理。坚决打赢保卫抚仙湖雷霆行动百日攻坚战，克期完成突出问题整改，开展第二阶段整治工作。实施抚仙湖综合保护治理三年行动计划，扎实推进“八大行动”，加快一级保护区生态移民搬迁，完成湖滨带生态修复工程建设，确保抚仙湖稳定保持I类水质。加快抚仙湖流域47个山水林田湖草项目前期工作、开工率达80%以上，推进种植结构调整和休耕轮作，恢复面山植被3万亩，加快径流区绿色生态经济区建设。着力推进星云湖环湖截污治污、底泥疏挖及处置、农业面源污染治理，恢复健康生态系统，确保消除劣Ⅴ类水质。加大杞麓湖环湖截污、入湖河道生态治理、流域村落环境综合整治力度，确保水质持续好转。加快3个湖泊国家湿地公园试点建设。加快75个“十三五”规划项目实施，力争项目开工率达100%。制定抚仙湖保护条例实施细则，做好杞麓湖保护条例修订工作。健全完善河长制、湖长制，建立“一河一湖一策一台账”，实施信息化、精细化管理，创建示范河道。加强南盘江、元江流域水污染综合防治，主要河流断面水质达到功能要求。加大14个县级以上饮用水源地保护力度，完成东风水库大小矣资搬迁。

加强生态建设。完成玉溪市生态文明建设规划编制。健全完善15个自然保护区规划。推进森林玉溪建设，实施灾害防控保障计划，完成营造林13万亩、退耕还林4.8万亩、石漠化治理5.9万亩，治理水土流失面积122平方公里。加强野生动物栖息地、原生地监管。推进生态文明创建活动，澄江创建为省级生态文明县、7个乡镇创建为生态文明乡镇。

提升环境监管能力。限时完成省环境保护督察发现问题整改。认真开展第二次全国污染源普查。加快实施大气污染防治行动，着力治理工业企业污染，全面实施机动车排气污染检验，加快淘汰黄标车老旧车，实行“绿色施工”，加强扬尘治理，提高保洁水平，确保中心城区和县城空气质量稳步提升。做好第三次全国土地调查，开展土壤污染状况详查，建立污染地块开发利用负面清单，确定重点监管企业名单及国控监测点，加快土壤污染治理与修复。推行清洁生产，强化重点企业行业监管，完成主要污染物总量减排目标任务。完善环境监测预警体系，健全环保信用评价和信息强制性披露制度。实行环境执法联动，严查重处违法行为。

（十）强化社会建设，努力保障和改善民生

提升就业和社会保障水平。抓好重点群体就业工作，新增城镇就业2.65万人，帮助7950名就业困难人员实现就业。进一步加大投入，不断提高社会保障水平。完成红塔区殡仪馆异地搬迁，全市辖区纳入火化区，确保火化率达100%、骨灰全部入公墓。

优先发展教育事业。出台全面加强教师队伍建设的实施意见。实施第三轮学前教育三年行动计划，推进办园体制改革，完成“全面改薄”和34所幼儿园、56所小学、9所初中新建改建及设备购置，实施高中质量提升计划，进一步提高义务教育办学水平。启动红塔区义务教育优质均衡发展试点，中心城区新增幼儿园学位520个、高中学位800个。解决好学前教育及高中就学难、中小学课外负担重、大班额、择校热等问题。加快推进职教园区、玉溪师范学院等4个教育补短板项目建设。强化校园安全管理，确保师生安全。

推动文化持续繁荣。强化意识形态工作，坚持正确舆论导向，大力弘扬社会主义核心价值观。持续建设聂耳音乐之都，加快文化广播影视传媒中心建设。加强文艺队伍建设和文化交流，打造文艺精品，发展文化产业。实施文物保护工程，加强文物古迹、古镇名村、名人故居及文化自然遗产保护。改扩建2个县级文化馆，建成5个乡镇文化站，基本实现全覆盖。

加快健康玉溪建设。出台公立医院改革国家示范城市建设工作方案，抓好全国健康城市试点。做好医养结合试点，启动市康养产业园、中山大学澄江教学医院建设，推进市中医医院扩建、妇产医院迁建前期工作，完成市医院改扩建和儿童医院建设，改扩建2个县级医院，建设8个标准化村卫生室。加快智慧健康养老产业发展。广泛开展全民健身运动，进一步加强体育工作，做好承办省第十六届运动会筹备工作，积极备战省第十五届运动会和第十届民运会。

提高社会治理水平。全面推进依法治市，深入实施“七五”普法。加强宗教事务管理，抓好第二轮“十县百乡千村万户”示范创建，深入实施民族团结进步示范工程。落实安全生产责任，加强基层安全生产网格化监管，提升食品药品安全保障水平，坚决防范重特大公共安全事故。开展矛盾纠纷大排查大调处活动，推进市县乡村四级综治中心建设，健全社会治安防控、社会心理服务和城乡社区治理体系，提高社会治理社会化、法治化、智能化、专业化水平。加强网络安全监管，开展扫黑除恶专项斗争，严厉打击各类违法犯罪活动，巩固“长安杯”创建成果，维护社会和谐稳定。

进一步做好防灾减灾、外事侨务、保密、档案、红十字、关心下一代等工作。切实保障好妇女儿童、老年人、残疾人合法权益。支持好工会、共青团、妇联等群团组织发展。推进军民融合深度发展，扎实抓好国防动员、国防教育、人民防空、民兵预备役、双拥共建、优抚安置等工作。

继续办好十件惠民实事。

各位代表！新时代新征程对政府自身建设提出了新的更高要求。我们将以不忘初心的信念、开拓创新的举措、务实清廉的作风，努力建设人民满意的政府，切实提高政府的公信力、执行力。

提高政治站位，全面从严治政。深入学习贯彻党的十九大精神，增强“四个意识”，坚定“四个自信”，坚决维护习近平总书记在党中央和全党的核心地位，坚决维护党中央权威和集中统一领导，坚决贯彻落实党中央国务院、省委省政府和市委各项决策部署。深入开展“不忘初心、牢记使命”主题教育，把全面从严治党要求落实到从严治政之中，加强思想政治建设，严守政治纪律和政治规矩，切实做到忠诚干净担当。加强班子建设，着力增强政府党组的执行力、凝聚力、战斗力。

严格依法行政，建设法治政府。自觉接受人大及其常委会法律监督、工作监督和政协民主监督，广泛听取各民主党派、工商联、无党派人士和人民团体的意见建议，认真办理人大代表建议和政协提案。严格落实法治政府建设实施方案，严守重大行政决策程序，严格执行重大行政决策责任追究制度，严格规范公正文明执法，健全政府法律、决策咨询顾问制度，提高政府决策科学化、民主化、法治化水平。

坚持务实创新，建设效能政府。开展“转作风、提效率、促落实”工作落实年活动，作为“不忘初心、牢记使命”主题教育的重要内容。发挥市政府领导班子垂范引领作用，强化公务员队伍管理，推行末位交流制度，解决部分公职人员不思进取、按部就班、互不协调、中间梗阻等问题。建立健全激励机制和容错纠错机制，完善综合考评办法，加强部门绩效管理，着力营造良好干事创业环境，让有信念、有思路、有激情、有办法的干部甩开膀子干。建立大督查机制，落实敬业有功、怠业必惩各项规定，坚决惩治庸懒散混。

坚守纪律底线，建设廉洁政府。认真落实党风廉政建设主体责任和“一岗双责”。不折不扣落实中央八项规定和实施细则精神以及省、市委实施办法，坚持不懈纠正“四风”。强化监管，推进审计全覆盖，加强农村集体公共资产管理，管好用好扶贫、低保、棚改、医保等资金，坚决整治侵害群众利益的不正之风，坚定不移惩治腐败。

各位代表！玉溪发展已经站在新的历史起点上，跨越式发展的号角已经吹响，美好蓝图已经绘就。让我们紧密团结在以习近平同志为核心的党中央周围，在省委、省政府和市委的坚强领导下，不忘初心、继续前进，不驰于空想、不骛于虚声，勇于担当、苦干实干，全力履行好新时代赋予的新使命，奋力谱写好中国梦的玉溪篇章！
